# Health Tract, No. 239

**Published:** 1866-05

**Authors:** 


HEALTH TRACT, No. 239.

### DOMESTIC CLEANLINESS.

On one occasion sickness prevailed in a family, which failed to obtain relief from any of the various remedies administered, but upon one of the panes of glass being broken and not repaired, an immediate improvement became apparent, resulting in ultimate good health.

In a fishing town in Cornwall, England, some of the houses in the narrower streets were in such a filthy condition from the negligence of those who occupied them, that the sanitary inspector considered it necessary to require the inmates to move into an open field, many of whom were already sick of cholera; they immediately began to improve; meanwhile the houses were cleaned out, swept, washed, and then thoroughly whitewashed; but no sooner had the families moved in, than the disease began to spread and assume a more malignant character; the people were again removed to the field, to sleep and cook and eat in the open air, when the same prompt improvement in their condition was manifested. It seemed that although the houses were cleansed, the yards and gardens around had been the recipients of offal of various kinds so long, that the very earth was saturated with the elements of disease.

Cases are recorded in standard medical books, where whole families have been stricken with disease in a few days; and examination discovered that the house drains had given away, and emptied themselves partially into the well from which the family derived all its supplies of drinking and cooking water. This was also the case in two English prisons, causing within a day or two, an epidemic dysentery throughout the establishment, which immediately ceased on a supply of better water.

The difference between cleanliness and the want of it, about a house, is demonstrated in some of the model lodging houses in London, standing in the midst of unhealthful surroundings; for in these houses the number of deaths is just half as many as in the immediate neighborhood.

It may be well to know what is an excess of sickness or death in any locality. In the Isle of Wight, one of the healthiest places on the globe, in a promiscuous population, for every thousand persons, fourteen died in the course of a year, from the ordinary sicknesses of humanity. In the model lodging houses, above referred to, about thirteen die out of a thousand, annually; while there are twenty-seven deaths among the surrounding inhabitants. In some portions of the British Army, where sanitary officers are scientific and conscientious men, only nine persons in a thousand, died annually; and in some of the best regulated prisons, in England, the death rate has been reduced to five in a thousand, a year, but in these last instances there are no children. In ordinary cases of soldier or prison-life, in what may be called "a standing army" or barrack life, not more than twenty persons in a thousand should die in a year, because in one sense they have nothing to do, but to keep their persons and habitations most perfectly clean.

In England twenty-two persons die annually out of each thousand; in the United States twenty-four. For each person who dies twenty-eight are sick. It is estimated that each death is equivalent to one person being sick for two years. Two hundred years ago eighty persons died out of a thousand annually in London, one hundred years ago fifty; and now, twenty-two, showing clearly, that as the intelligence of a people increases, as to the laws of health, sickness and death proportionably abate.

